# Comparative Meta-Analysis of Minimally Invasive and Conventional Approaches for Caries Removal in Permanent Dentition

**DOI:** 10.3390/medicina60030402

**Published:** 2024-02-27

**Authors:** Diego González-Gil, Javier Flores-Fraile, Vicente Vera-Rodríguez, Andrea Martín-Vacas, Joaquín López-Marcos

**Affiliations:** 1 Dental Clinic Faculty of Medicine, Surgery Department, University of Salamanca, 37007 Salamanca, Spain; diegoggil@usal.es (D.G.-G.); jflmarcos@usal.es (J.L.-M.); 2 TUFTS Dental School, Boston, MA 02155, USA; vicente.vera_rodriguez@tufts.edu; 3 Faculty of Dentistry, Alfonso X El Sabio University, 28691 Madrid, Spain; amartvac@uax.es

**Keywords:** selective caries removal, incomplete caries removal

## Abstract

*Background and Objectives:* Addressing deep carious lesions poses significant challenges in daily dental practice due to the inherent complexity of their treatment. Traditionally, complete removal of carious tissues has been the norm, potentially leading to pulp tissue exposure and subsequent pulpitis. In contemporary dentistry, there is a growing preference for minimally invasive techniques, such as selective removal, offering a more conservative approach with enhanced predictability and success rates. *Materials and Methods:* Our study commenced with a comprehensive systematic review. After that, we performed a meta-analysis focused exclusively on randomized controlled trials involving permanent dentition. Our investigation incorporated seven selected articles, which scrutinized success rates and the incidence of pulp exposure in minimally invasive techniques (MIT) versus conventional techniques (CT). Statistical analysis employed U Mann–Whitney and Wilcoxon tests to interpret the results. *Results:* Although the difference did not reach statistical significance, MIT demonstrated marginally superior success rates compared to CT. Furthermore, MIT exhibited a lower percentage of pulp exposure when contrasted with CT. However, due to the limited sample size, statistical significance for this difference could not be established. *Conclusions:* Minimally invasive techniques for caries removal emerge as a conservative and promising approach to safeguard pulp tissues in comparison to conventional techniques. The need for additional randomized controlled trials is emphasized to unequivocally establish the superior success rates of these procedures over their conventional counterparts.

## 1. Introduction

Dental caries is a biofilm-mediated, diet modulated, multifactorial, dynamic disease resulting in mineral loss of dental hard tissues [1]. Nowadays, this disease is widespread throughout the world, affecting billions of teeth [2,3,4]. When caries produces very deep lesions in dentin, dental pulp can be damaged, compromising its vitality. Over the years, dentists have faced the challenge of knowing how much dentin to remove or preserve in order to perform a proper treatment [5,6,7,8]. During the 19th century, G.V. Black suggested that carious tissue should be removed until sound physiological dentin was reached, although this procedure may lead to pulp exposure. Almost a century later, Fusuyama improved caries removal techniques by recognizing two different types of dentine: inner demineralized zone or affected dentin and outer contaminated or infected dentine [9,10]. The inner zone presents a firm consistence, as there is a little resistance while removing it. As this zone is susceptible to remineralization, it should be respected and maintained. The outer zone presents soft dentin as it is highly contaminated, and should be completely removed during our restorative procedures [11].

Traditionally, complete caries removal procedures such as stepwise technique have been used to remove deep caries in a non-selective way. Stepwise technique consists of the elimination of both firm and soft dentin in deep caries removal. This procedure may cause pulp exposure and excessive removal of tooth structure, and it is performed in two different visits by placing a temporary restoration between appointments. This conventional method presents some disadvantages; for instance, a high risk of irreversible pulp damage, which results in higher costs to the patient, or an increase in office visits [6,12,13].

Nowadays, minimally invasive dentistry should be a guide mark to restore dental caries and there are new procedures that offer advantages over conventional methods. Although selective procedures are well documented in the literature, with respect to deciduous teeth, there are few investigations about these techniques in permanent dentition [14]. Since both dentitions present differences, such as size of teeth or regenerative potential, they should be studied separately. Selective caries removal to soft or firm dentine is less invasive than stepwise technique, and it offers great results. This method is also known as incomplete caries removal technique, and it pretends to respect pulp tissues by not eliminating soft dentin near the pulp chamber [15,16,17,18,19,20,21,22]. While removing decay, procedures such as caries dyes or laser fluorescence can be used to perform a proper selective technique. Caries detector dyes stain outer and inner zones differently, in order to distinguish them. However, this procedure may lead to excessive tissue removal, as it is not accurate in assessing the amount of contamination present in each zone [23,24]. Another product that is used during selective caries removal is Cariosolv, which is a gel that removes contaminated dentin, respecting demineralized tissues. This procedure, when combined with a microscope, can be a great alternative to restore deep caries [25,26,27,28]. On the other hand, the DIAGNOdent pen uses fluorescence technology for the detection of bacterial porphyrins and it provides useful information about dentin contamination [29]. Another effective procedure using laser fluorescence during selective caries removal is the FACE device, which consists of an orange-red laser that allows recognition of highly contaminated areas [30,31]. Tactile evaluation of tissue hardness should be taken into account along with laser fluorescence and caries detector dyes as effective methods to achieve a proper selective treatment [10,32,33].

While performing selective caries removal, it is important to follow a great bonding protocol to obtain successful long-term restorations [12,34,35]. Only sound dentin allows an adequate bond strength, as inner and outer dentin present high percentages of bond strength loss from 25% to 66%, respectively. This important decrease in values is due to the great demineralization in these carious zones [36,37,38]. A key point to accomplish a suitable bond strength is to create a peripheral seal zone 1 to 3 mm wide, free from soft dentin. Caries detector dyes and fluorescence techniques will help us to confirm the absence of contaminated dentin outside our peripheral seal zone. Highly infected dentin inside our seal area should be eliminated without exposing pulp chamber. Regarding the bonding system, it is recommended to use a three-step total etch or a mild two-step self-etching in order to obtain a great bond strength during selective caries removal [9,10]. With this investigation, we pretend to compare success rates in minimally invasive techniques and conventional procedures in order to know which one offers better results, as well as studying pulp exposure in both techniques to evaluate its influence in success rates.

## 2. Materials and Methods

### 2.1. Study Desing

We performed a systematic review of randomized controlled trials that studied selective caries removal techniques and showed results of pulp exposure and success rates in permanent dentition, including articles from the period from January 2008 to December 2022, covering only articles published in English. After that, we conducted a meta-analysis with those articles that met our study requirements. We carried out a study selection according to the Preferred Reporting Items for Systematic Review and Meta-Analyses (PRISMA) guidelines for reporting systematic reviews. This investigation is property registered with in Prospero database with this code: CRD42023390509 Prospero Register Code.

The search strategy was conducted using the population, intervention, comparison, and outcome (PICO) framework based on the following question: “is the success rate of selective caries removal techniques better than conventional ones?”

### 2.2. Inclusion Criteria

We have only included randomized controlled trials that compared selective and non-selective techniques of caries removal in permanent teeth, as well as articles written in English and with the full text available.

### 2.3. Exclusion Criteria

**Figure 1:**
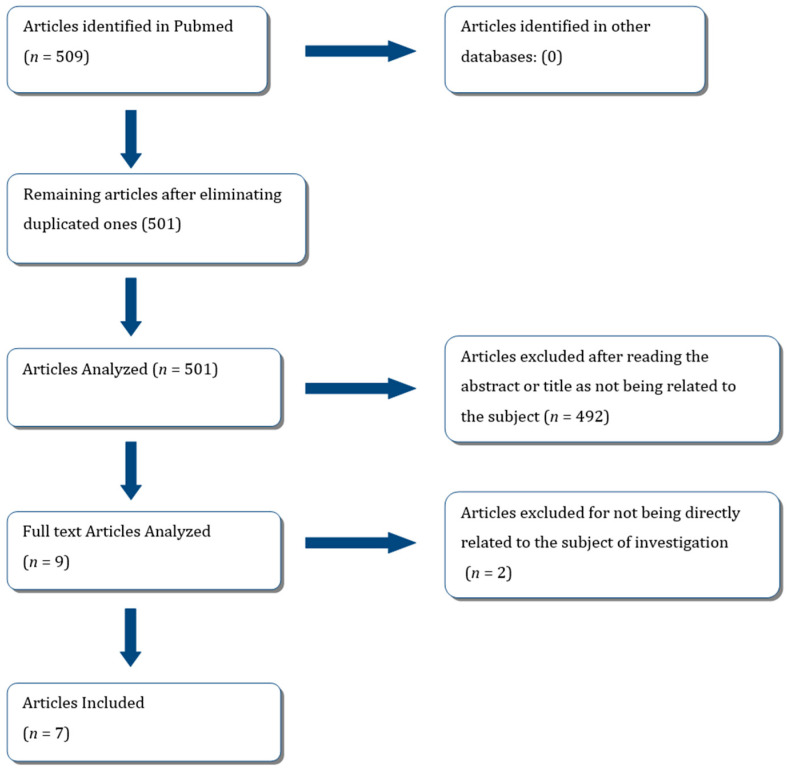
Flowchart showing systematic review.

After conducting the systematic review and reviewing titles and abstracts of selective procedures, we observed that the majority of investigations were focused on primary dentition. Consequently, all articles that examined techniques in primary dentition were excluded, as well as unfinished trials. This process is illustrated in the flowchart (Figure 1).

### 2.4. Variables

After obtaining values of each kind of technique, we established a comparison between them in order to know which one offers better results. All data was analyzed statistically.

#### Quality Assessment of the Included Studies

**Figure 2:**
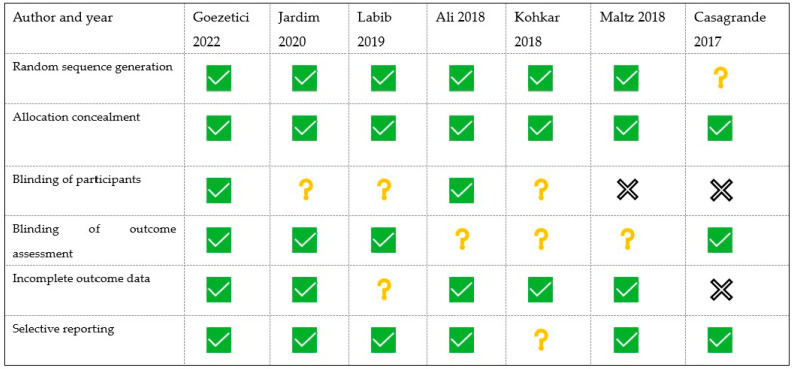
Quality assessments of included studies: 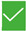 indicates low risk of bias; 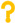 represents an unclear risk of bias; 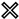 represents a high risk of bias [39,40,41,42,43,44,45].

The risk of bias of each included study was estimated using the Cochrane risk bias assessment tool, as it is represented in Figure 2. Most of these studies present a low risk of bias, especially those that are more recent. Studies such as Jardim, Labib, or Maltz focus on a type of procedure that must be performed in two appointments, making it impossible to blind patients. Casagrande’s study is retrospective, which makes some of the risk of bias domains challenging to measure. However, random sequence generation, allocation concealment, blinding, and reporting are well performed in these investigations.

### 2.5. Statistical Analysis

With respect to statistical analysis, we included normality test of quantitative variables in order to apply parametric or no-parametric tests. If the data number is greater than 50, we considered using a Kolmogorov–Smirnov test; or a Shapiro–Wilk test, in cases where this value is less than 50. We performed a comparison of mean values of distribution in quantitative variable of each group, that is determined by qualitative variable. When this variable presents two categories, Student’s *t* test should be used, but in this case, we applied the Mann–Whitney U non-parametric test, as there is a small sample. We used paired sample test to prove if it is possible to accept, with 95% confidence, that there is a statistical difference between variables. In this investigation, there is a small sample, so we used the Wilcoxon non-parametric test. All statistical test were applied with a confidence level of 95% by using SPSS version 26.0 software.

### 2.6. Resources

#### Bibliographical Resources

Medical Database Pubmed-Medline was consulted, and social media ResearchGate was used as a complement in order to obtain some full-text articles.

The key words used were “selective caries removal” and “incomplete caries removal”. A cross-search was also performed using these terms: “selective caries removal and permanent”.

## 3. Results

After performing a complete review in the literature, only seven articles were included in this metanalysis, as there are very few randomized trial controls that study the comparison of different caries removal procedures in permanent dentition. Besides this, there is a lack of homogeneity in conventional and minimally invasive methods as we had to mix similar procedures with variations in their protocols. In this manner, we categorized these techniques into two types: minimally invasive techniques (MIT), which encompass selective caries removal to soft dentin, self-limiting techniques, and incomplete caries removal. On the other hand, within conventional techniques (CT), we included selective caries removal to firm dentin, step-wise techniques, and complete caries removal.

**Figure 3:**
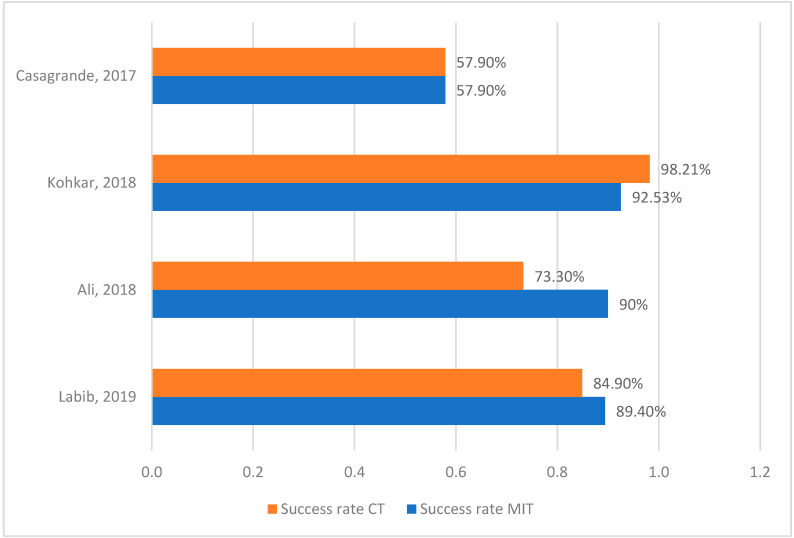
Success rate comparison [41,42,43,45].

**Figure 4:**
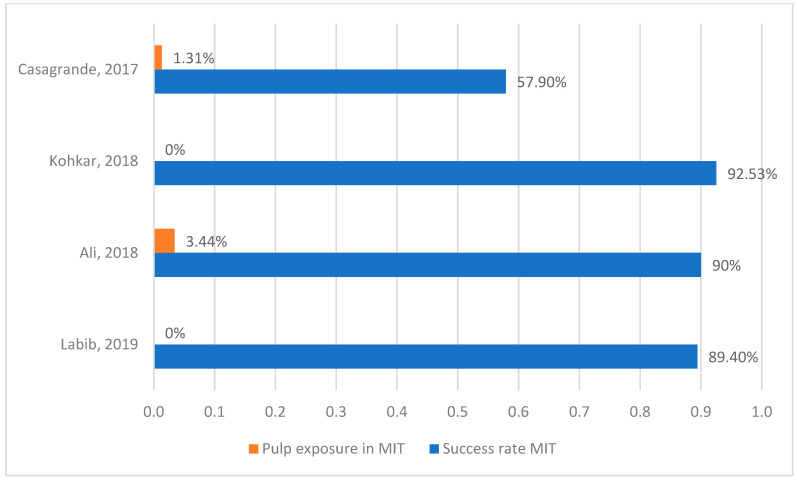
Pulp exposure and success rate in MIT [41,42,43,45].

**Figure 5:**
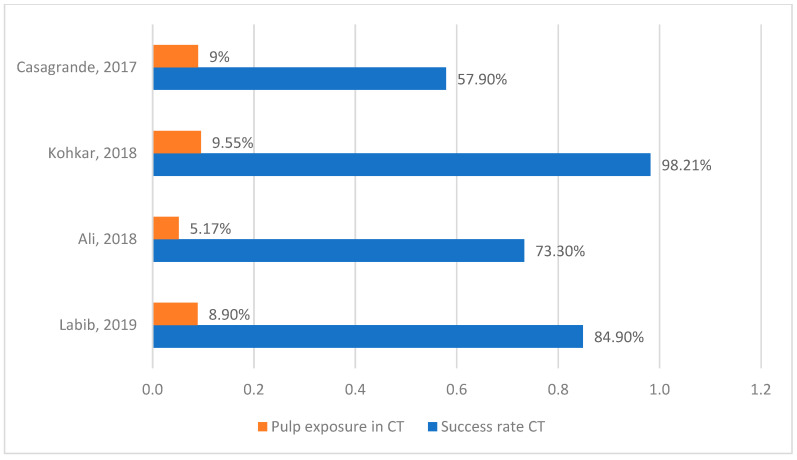
Pulp exposure and success rate in CT [41,42,43,45].

The results of the metanalysis are represented in Table 1. This table shows the sample and the follow-up of every investigation, as well as a brief description of each technique. Also, the percentages of success rate and pulp exposure are represented in the articles that measured these values. Success rates comparisons of minimally invasive techniques and conventional techniques are shown in Figure 3. Figure 4 and Figure 5 show pulp exposure rates in MIT and in CT. Not all investigations studied pulp exposure, so there is a lack of figures in those columns. The Mann–Whitney Test indicated no significant difference between both techniques, with a result of 0.2 (*p*-value > 0.05). The Wilcoxon Test yielded a value of 0.0, which is in close proximity to our significance threshold (*p*-value = 0.068).

## 4. Discussion

While performing this meta-analysis, we realized how scarce the literature is regarding the comparison between selective and non-selective removal procedures in permanent dentition. Although there are several randomized control trials on primary teeth [46,47,48,49], it is very difficult to find these types of investigations on permanent teeth. Furthermore, some of these studies on permanent teeth are still unfinished [50,51]. Another complication during this investigation was the lack of homogeneity of all techniques, which forced us to include similar procedures that presented variations in their protocols in the same study group [39,40,41,42,43,44,45]. We believe that further research should be conducted to investigate various techniques in order to establish a gold standard method that unifies proven successful protocols for the selective treatment of caries. A comprehensive exploration of these methods will contribute to the development of standardized techniques that guide our treatments in a minimally invasive direction, reducing trauma and costs for the patient.

Nowadays, minimally invasive dentistry, as well as biomimetic dentistry, has gained a lot of importance in our dental offices. However, when it comes to the treatment of caries, the majority of professionals still prefer to use conventional techniques, and there is limited documentation in the literature. According to this new treatment philosophy, we aim to be as conservative as possible with dental tissues. Specifically, when referring to caries removal, we should try to preserve the dental pulp and maintain the vitality of the affected tooth [2,3].

With this investigation, we aim to compare if minimally invasive caries removal techniques present better results than conventional ones and if pulp exposure that may occur while performing these procedures may influence their success. All the articles we analyzed present a comparison between a less invasive technique and another more invasive one, so we divided the procedure into two kinds: minimally invasive techniques and conventional techniques. In minimally invasive techniques (MIT), we included the following procedures: selective caries removal to soft dentin in one step [39,40,41,45], self-limiting technique [42] and partial caries removal [43,44]. All of these procedures are associated with conservative dentistry and demonstrate a high level of respect for pulp tissue. The self-limiting technique consists of an experimental protocol that combines the use of a clinical microscope with the chemomechanical removal of deep carious tissue using Cariosolv gel [42]. In conventional techniques (CT) we considered the following: selective caries removal to firm dentin [38], step-wise excavation [40,41,44] and complete caries removal [42,43,45]. Both selective caries removal to firm dentin and complete caries removal are too invasive as they do not respect affected dentin at the pulp wall. In these conventional procedures, all affected and infected dentin is removed from the cavity, even though pulp exposure may occur.

The step-wise procedure is based on the elimination of decayed tissue in two appointments. During the initial visit, carious dentin is excavated from the surrounding walls while maintaining affected dentin at the pulpal wall. Subsequently, a temporary seal is applied for 6 or 9 months, aiming to isolate microorganisms and control caries progression. In a follow-up visit, the cavity is reopened to complete the excavation, and the final restoration is placed [43]. Although this technique is more respectful to pulp tissues than complete removal procedures, it is not considered a minimally invasive procedure due to the duration of the temporary restoration and the increased number of dental appointments. As evidenced in the literature reviewed for this investigation, minimally invasive techniques yield excellent results within a shorter procedural time, sparing the patient from the need for two complex appointments. On the other hand, complete caries removal is a traditional technique that is less conservative and may cause harm to the pulp in many cases. By opting for selective caries removal, we can preserve pulp vitality, thereby avoiding the need for endodontic treatment, which is a significant benefit for the patient.

Initially, we compared the success rates of minimally invasive techniques (MIT) with those of conventional techniques (CT). Mean values in MIT (83.6%) are higher than CT ones [75.4%], indicating that selective techniques seem to be slightly more effective than conventional ones. By analyzing these results, it becomes evident that minimally invasive techniques are a superior alternative to conventional techniques when treating deep carious lesions. Not only do MITs exhibit higher success rates compared to CT, but they also offer multiple advantages to both professionals and patients. On one hand, MITs are less traumatic for the patient, preserving more hard tissue and maintaining pulp vitality. By avoiding endodontic treatment, our restorations become easier to perform and may have a longer lifespan when performed correctly. Additionally, in most cases, we can reduce the number of appointments and the overall duration of the treatment.

Another important factor to take into account is that the follow-up period in the studies is relatively short. Most studies have a follow-up period of 1 to 3 years, while only two articles have a follow-up of 5 years [40,44]. Therefore, it would be desirable to have more long-term investigations to compare success rates. Fortunately, there are some long-term studies that are currently underway and are expected to yield new and interesting results about minimally invasive procedures [50,51]. We anticipate incorporating these investigations into future analyses. After performing Mann–Whitney U to compare mean values between MIT and CT success rate, the results showed that there was not a statistically significant difference between these variables (*p*-value > 0.05).

Regarding pulp exposure percentages, minimally invasive techniques present a lower incidence of this complication compared to conventional procedures. It is conceivable that conventional techniques result in greater tissue removal. As a result, it is more probable that they will harm the dental pulp and compromise the success of our restorations. In our opinion, pulp exposure is a crucial aspect to consider when treating deep carious lesions. This characteristic should be assessed in every study to demonstrate the level of conservatism of a caries removal technique. New research should focus on the frequency of treatments leading to pulp exposure and establish a comparison between procedures. Unfortunately, only four out of the seven articles included in this meta-analysis reported rates of pulp exposure. [41,42,43,45]. While MIT presents 1.2% of pulp exposure, this value in CT is 8.1%; almost seven times higher than the previous one.

An important aspect that we needed to consider in our investigation was whether the rates of pulp exposure were influenced by the type of procedure used for caries removal. So, we analyzed both variables statistically using Wilcoxon non-parametric test, due to the small sample of this investigation. The results of this analysis showed that there was no significant difference between both techniques (*p*-value > 0.05) and therefore, pulp exposure was not influenced by the kind of procedure. However, the *p*-value is very close to our limit of significance (*p*-value = 0.068), and since our sample size is very small, it is acceptable to assume that if we could have included more studies in our meta-analysis, the difference could have been significant.

The current trend in dentistry is to orient our treatments in the most conservative way. Other areas, such as surgery or prosthesis, present new minimally invasive techniques that reduce trauma to the patient, as well as the duration of treatment. However, when it comes to caries removal, in most cases, a conventional treatment protocol is still implemented, consisting of eliminating all carious tissue without respecting pulp vitality. Minimally invasive caries removal techniques, such as selective treatments, are a great alternative to complete removal procedures, showing good results in success rates of restorations and pulp exposure rates. Adapting our restorative protocols to selective caries removal techniques allows us to offer significant benefits to patients without increasing the risks or economic and time costs. With regard to the materials needed to perform minimally invasive techniques, caries detector dyes and fluorescence devices are utilized for delineating the boundaries of our restorations, facilitating the establishment of a proper peripheral seal and ensuring correct bonding. However, these materials are not always precise, and often result in excessive tissue removal and overpreparation. Therefore, new techniques and devices should be designed to be as respectful and minimally invasive as possible [11,29,30]. If we are very careful with our bonding protocol and follow selective removal procedures, we can preserve affected dentin at the pulpal wall, controlling the progression of caries. After restorative procedure, It is important to maintain a close follow-up to observe how pulp tissue responds to minimally invasive techniques while measuring pulp vitality.

In our opinion, selective removal procedures should be further investigated with larger samples in multicentric and long-term studies to obtain a gold-standard treatment protocol that could be as respectful as possible to pulp tissues, as well as very conservative with hard tissues. This way, with reliable and reproducible results, professionals worldwide can opt for selective procedures rather than conventional ones.

## 5. Conclusions

1. Using minimally invasive techniques for caries removal presents slightly better success results than conventional techniques, although this difference is not significant.
2. Minimally invasive techniques result in a lower percentage of pulp exposure than conventional ones, which may lead to improved success rates in those techniques.
3. More long-term RCTs in the permanent dentition are required to demonstrate whether there is a significantly reduced risk of pulp exposure and higher success in preserving pulp vitality when minimal invasive caries removal techniques are applied.

## Figures and Tables

**Table 1:** Articles meta-analyzed.

Study and Year	Sample	Follow-Up	Minimally Invasive Technique	vs.	Conventional Technique	Success Rate MIT	Success Rate CT	Pulp Exposure in MIT	Pulp Exposure in CT
Goezetici, 2022 [39]	113 restorations	2 years	Selective removal to soft dentin: an amount of carious tissue was left over the pulp		Selective removal to firm dentin: carious tissue was completely removed	96.7%	83.2%	-	-
Jardim, 2020 [40]	172 restorations	5 years	Selective removal to soft dentin: only disorganized dentin was removed		Stepwise excavation: two-step complete caries removal	79%	76%	-	-
Labib, 2019 [41]	106 restorations	1 year	Selective caries removal in one step		Stepwise excavation: two-step complete caries removal	89.4%	84.9%	0%	8.9%
Ali, 2018 [42]	85 restorations	1 year	Self-limiting technique: use of microscope combined with chemo mechanical excavation		Conventional technique: subjective removal to leathery dentin	90%	73.3%	3.4%	5.2%
Kohkar, 2018 [43]	123 restorations	1, 5 years	Partial caries removal: soft dentin was left over the pulp		Complete caries removal	92.5%	98%	0%	9.5%
Maltz, 2018 [44]	229 restorations	5 years	Partial Caries removal: selective caries removal in one session		Stepwise excavation: two-step complete caries removal	80%	56%	-	-
Casagrande, 2017 [45]	477 restorations	3 years	Selective caries removal		Complete caries removal in one session	57.9%	57.9%	1.2%	9%

## Data Availability

Data are contained within the article.
